# Enhancing genome-scale metabolic models with kinetic data: resolving growth and citramalate production trade-offs in *Escherichia coli*

**DOI:** 10.1093/bioadv/vbaf166

**Published:** 2025-07-12

**Authors:** Jorge Lázaro, Arin Wongprommoon, Jorge Júlvez, Stephen G Oliver

**Affiliations:** Department of Computer Science and Systems Engineering, University of Zaragoza, Zaragoza, 50018, Spain; Department of Biochemistry, University of Cambridge, Cambridge, CB2 1QW, United Kingdom; Human Genetics Programme, Wellcome Sanger Institute, Wellcome Genome Campus, Cambridge, CB10 1SA, United Kingdom; Department of Computer Science and Systems Engineering, University of Zaragoza, Zaragoza, 50018, Spain; Aragón Institute for Engineering Research, University of Zaragoza, Zaragoza, 50018, Spain; Department of Biochemistry, University of Cambridge, Cambridge, CB2 1QW, United Kingdom

## Abstract

**Summary:**

Metabolic models are valuable tools for analyzing and predicting cellular features such as growth, gene essentiality, and product formation. Among the various types of metabolic models, two prominent categories are constraint-based models and kinetic models. Constraint-based models typically represent a large subset of an organism’s metabolic reactions and incorporate reaction stoichiometry, gene regulation, and constant flux bounds. However, their analyses are restricted to steady-state conditions, making it difficult to optimize competing objective functions. In contrast, kinetic models offer detailed kinetic information but are limited to a smaller subset of metabolic reactions, providing precise predictions for only a fraction of an organism’s metabolism. To address these limitations, we proposed a hybrid approach that integrates these modeling frameworks by redefining the flux bounds in genome-scale constraint-based models using kinetic data. We applied this method to the constraint-based model of *Escherichia coli*, examining both its wild-type form and a genetically modified strain engineered for citramalate production. Our results demonstrate that the enriched model achieves more realistic reaction flux boundaries. Furthermore, by fixing the growth rate to a value derived from kinetic information, we resolved a flux bifurcation between growth and citramalate production in the modified strain, enabling accurate predictions of citramalate production rates.

**Availability and implementation:**

The Python code generated for this work is available at: https://github.com/jlazaroibanezz/citrabounds

## 1 Introduction

### 1.1 Kinetic and constraint-based models

Metabolic models are mathematical representations of the metabolic reactions of an organism. Such models have been used to analyze key features of the organism, e.g. gene essentiality (Caserta *et al.* 2012) and growth, and to develop new biotechnological milestones (Gu *et al.* 2019, Passi *et al.* 2021, Enuh *et al.* 2022). Two popular modeling approaches for metabolism are kinetic models (Chassagnole *et al.* 2002) and constraint-based models (Varma and Palsson 1994, Orth *et al.* 2011). While both approaches specify the stoichiometry of metabolic reactions, kinetic models provide detailed reaction rates based on metabolite concentrations and reaction kinetics, whereas constraint-based models only specify flux bounds based on stoichiometry and mass conservation constraints. Kinetic and constraint-based models are analyzed in different ways: integrating differential equations in kinetic models produces precise deterministic time trajectories of both the concentration of metabolites and the reactions’ rates, while constraint-based models can be analyzed as linear programming problems to produce a potential set of optimal fluxes over the reactions.

Kinetic models are difficult to develop because modeling and validating the kinetics of a metabolic reaction require a large amount of information (Foster *et al.* 2021). Furthermore, the integration of large non-linear ordinary differential equations might be computationally complex. Therefore, such models are relatively small and tend to focus on central carbon metabolism or a particular pathway (Saa and Nielsen 2017). On the other hand, constraint-based models only specify the stoichiometry and the flux bounds of reactions. Given the simplicity of the constraints that they incorporate, i.e. just reaction stoichiometries, it is relatively easy to build large constraint-based models; consequently, genome-scale constraint-based models exist for several organisms (Gu *et al.* 2019, Fang *et al.* 2020). A disadvantage of these models is their lack of precision (Durot *et al.* 2009, Mao *et al.* 2015, Shaw and Cheung 2021) and although genome-scale metabolic models have proven useful to predict specific phenotypes (e.g. growth rates under different conditions), the constraints they usually include are not enough to accurately predict intracellular fluxes (Yeo *et al.* 2020, Schroeder *et al.* 2024). Moreover, estimating the values of competing objective functions, such as biomass and product formation, is not straightforward in constraint-based models.

Competing objectives, such as biomass production and metabolite synthesis, often lead to unrealistic predictions in Flux Balance Analysis (FBA) models due to the absence of biologically grounded constraints that account for resource trade-offs. Some current methods impose biologically realistic limitations, creating natural trade-offs between growth and production, and yielding more accurate predictions of cellular behavior. Approaches like GECKO (Domenzain *et al.* 2022) and sMOMENT (Bekiaris and Klamt 2020) address this by imposing enzyme capacity limits, but they rely on simplified kinetics and often incomplete or estimated $k_{\mathrm{cat}}$ values. In our work, we resolve these trade-offs using detailed enzymatic data by simulating a kinetic model of central carbon metabolism to steady state. This model incorporates complex rate laws, regulatory interactions, and metabolite feedback mechanisms that are typically missing in standard genome-scale methods. The resulting fluxes reflect realistic system dynamics and are used to constrain the genome-scale model, even when only a subset of reactions is kinetically characterized. This enables the integration of condition-specific and regulation-aware information without requiring full kinetic coverage. As a result, our method supports a biologically plausible behavior, including non-zero growth under competing objectives. Furthermore, it is less data-intensive than alternatives such as RBA (Goelzer and Fromion 2011) or ME-models (Lloyd *et al.* 2018).

Previous studies have attempted to bridge the gap between kinetic and constraint-based models. Berzins *et al.* (2022) examined the production of docosahexaenoic acid from dinoflagellate *Crypthecodinium cohnii* by evaluating whether fluxes from independently simulated kinetic and constraint-based models were in agreement. However, this study does not integrate information from kinetic model simulations to enhance the constraint-based model, or vice versa. The work in Moulin *et al.* (2021) reviews methods to combine kinetic and constraint-based models and provides a small example based on first principles. However, such an example only has six reactions, substantially less than the number of reactions in recent kinetic and constraint-based models; therefore, applying their method to such models will be computationally expensive due to the exponential increase derived from different combinations of parameter values. The study further proposes narrowing the solution space, but this does not address the pathway branching that can arise when there are competing objective functions in a constraint-based model. In contrast to previous approaches, the present work simulates an independent kinetic model and uses the resulting fluxes to constrain reactions in a genome-scale model. To achieve this, a mapping was established between the reactions of the kinetic model and those of the constraint-based model. The resulting constraint-based model narrows the range of potential steady-state fluxes, yielding more realistic values and resolving potential bifurcations of fluxes.

### 1.2 Citramalate: an industrially relevant metabolite

Citramalate is a dicarboxylic acid not commonly encountered, but it is present in some bacteria, such as *Methanocaldococcus jannaschii* and fungi. It has been found out that it is an intermediate in certain metabolic pathways, including the biosynthesis of isoleucine in bacteria (Sugimoto *et al.* 2021). *M. jannaschii* expresses the gene *CimA3.7*, encoding the citramalate synthase enzyme (EC: 2.3.1.182) to produce citramalate. This enzyme catalyzes the reaction in which one molecule of acetyl-CoA, one molecule of pyruvate, and one molecule of water react to produce one molecule of (3R)-citramalate, one molecule of CoA, and a proton (UniProt Consortium 2018), see Equation (2).

Citramalate offers a sustainable alternative to fossil fuels for producing industrially important compounds. It can be converted into methacrylic acid, a precursor for methyl methacrylate (MMA), which is used to produce poly MMA (pMMA), widely applied in dentistry, electronics, and paints. This approach reduces reliance on petroleum-based processes, which harm ecosystems through greenhouse gas emissions and hazardous waste generation (Sugiyama *et al.* 2009).

Genetically modified organisms, such as *E. coli*, can produce citramalate at an industrial scale in bioreactors. *E. coli* is ideal due to its rapid biomass production and ease of genetic modification, yielding 0.48 grams of citramalate per gram of glucose under specific conditions (Webb *et al.* 2018). Mathematical modeling of this process could optimize bioreactors and replace fossil fuel-derived MMA precursors.

### 1.3 Aims and objectives

We aim to bridge the gap between kinetic and constraint-based models. In particular, we showed how to enhance a genome-scale constraint-based model with kinetic information obtained from a kinetic model to obtain a more realistic constraint-based model. We enriched the constraint-based model through the following steps: (i) map reactions between the kinetic and the constraint-based models; (ii) compute fluxes of the kinetic model in steady-state simulations; and (iii) translate the bounds to the constraint-based model. To help map and translate flux bounds, we represented reactions graphically as Petri nets (Heiner *et al.* 2008). To assess the enhanced constraint-based model, FBA and Flux Variability Analysis (FVA) (Orth *et al.* 2010) were used.

We demonstrated our approach using a kinetic (Millard *et al.* 2017) and a constraint-based model (Orth *et al.* 2011) of *E. coli*. Concerning the FVA, we examined the number of reactions with increased or decreased variability (given by the flux variability value) after incorporating kinetic constraints into the original constraint-based model. We showed that enhancing the constraint-based model with kinetic information results in the reallocation of reaction fluxes due to the incorporation of tighter constraints, as well as the activation of reactions that initially had no flux. Additionally, we extended these models to simulate the production of citramalate. An undesirable feature of the extended constraint-based model is a bifurcation that allows the model to fully direct the nutrient flux either towards the production of biomass or towards the production of citramalate. Thus, maximizing growth rate leads to null citramalate production, and similarly, maximizing citramalate production results in an unrealistic zero growth. We showed that enriching this constrained-based model with kinetic information and fixing the growth rate solves the split of pathways problem, resulting in a more realistic value of citramalate production efficiency.

## 2 Materials and methods

### 2.1 Kinetic model

We used the *kinetic model* of *E. coli* introduced in Millard *et al.* (2017). This model captures the main central carbon pathways and accounts for 68 reactions and 77 metabolites which are located in 3 compartments: environment, periplasm, and cytoplasm. The model represents glucose-limited conditions and is expressed as a set of ordinary differential equations—see Millard *et al.* (2017) for details on the development of the model. Out of the 68 reactions, 48 include $V_{\max}$, the maximum flux that a reaction can carry under specific conditions, as a parameter. Of these reactions, 41 correspond to enzyme-catalyzed reactions (the remaining 7 reactions with an assigned $V_{\max}$ are exchange reactions, the growth reaction, or the ATP_MAINTENANCE reaction, and hence, cannot be attributed to a particular enzyme). The $V_{\max}$ parameter can be perturbed to account for different cellular conditions arising from different enzyme concentrations, as expressed by Equation (1):

(1) $$V_{\max}=k_{\mathrm{cat}}\cdot [E]$$

where *[E]* is the enzyme concentration and $k_{\mathrm{cat}}$ is the turnover number.

The kinetic model was analyzed by integrating its set of differential equations until the steady state was reached, evidenced by infinitesimal changes in substrate concentrations, usually after a simulation time of 3600 s.

### 2.2 Constraint-based model

A *constraint-based model* is a tuple {R, M, S, L, U}, where R is the set of reactions, M is the set of metabolites, $S\in R^{\left|M|\times |R\right|}$ is the stoichiometric matrix, and $L,U\in R^{\left|R\right|}$ are the lower and upper flux bounds, respectively, of the reactions.

This study used the constraint-based *E. coli* model iML1515, described in Orth *et al.* (2011). The model includes 2712 reactions, 1516 genes, and 1877 metabolites across three compartments: extracellular space, periplasm, and cytoplasm. Each reaction in the model specifies reactants, products, and fixed flux bounds.

### 2.3 Petri nets to represent metabolic networks

Petri nets are a popular modeling formalism for dynamical systems (Murata 1989, Silva 1993). A Petri net is a directed bipartite graph with two types of vertices: *places*, which are depicted as circles, and *transitions*, which are depicted as rectangles.

Petri nets can be used to graphically represent metabolic networks (Heiner *et al.* 2008). In such a representation, metabolites are modeled by places and reactions are modeled by transitions. A metabolite that is a reactant in a reaction is connected with an arc from the metabolite to the reaction. Similarly, a metabolite that is a product in a reaction is connected with an arc from the reaction to the metabolite.

**Figure 1.**
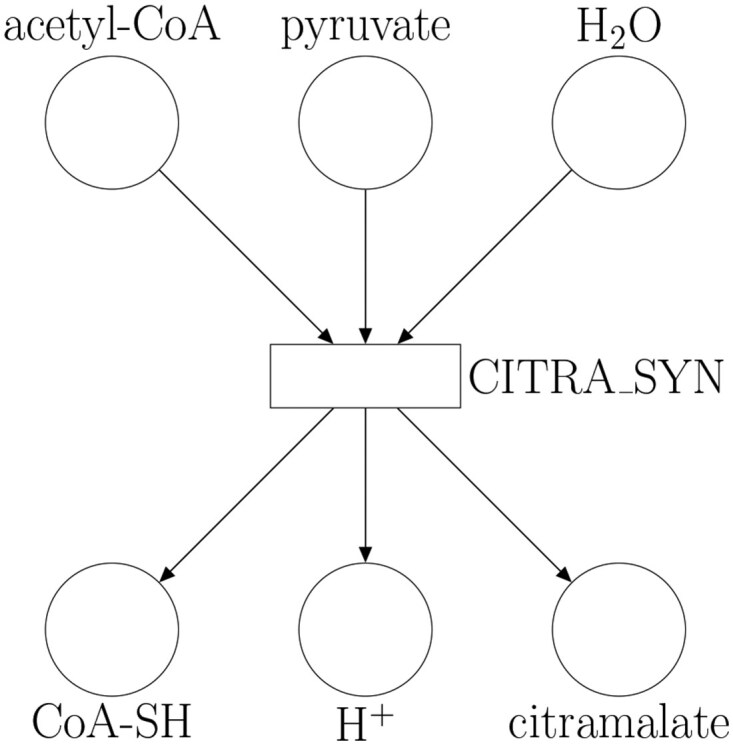
Petri net modeling the citramalate synthesis reaction specified in Equation (2). Metabolites are modeled by places, which are depicted as circles, and reactions are modeled by transitions, which are depicted as rectangles.

As an example, the Petri net in Fig. 1 models the reaction for citramalate synthesis:

(2) $$\begin{array}{c} \mathrm{acetylCoA}+\mathrm{pyruvate}+H_{2}O\to \mathrm{CoASH}+H^{+}+\mathrm{citramalate} \end{array}$$

Metabolites acetyl−CoA, *pyruvate*, and $H_{2}O$ are reactants so they are modeled as input places of transition CITRA_SYN, which models the reaction, and metabolites CoA−SH, $H^{+}$, and *citramalate* are products, modeled as output places. A comprehensive Petri net is obtained once all the metabolites and reactions in the metabolic network are modeled with Petri net elements. Representing reactions as Petri nets helps us transfer flux bounds between kinetic models and constraint-based models.

### 2.4 FBA and FVA simulations

FBA calculates steady-state metabolic fluxes in a network by optimizing an objective function (e.g. biomass production) subject to mass balance constraints. It assumes that the system is at a steady state, and the goal is to find a set of optimal fluxes that maximizes or minimizes a given objective function. FBA can be computed via the following linear programming problem:

(3) $$\begin{array}{c} \begin{array}{c} \mathrm{Max}\;c^{T}\cdot v\\ S\cdot v=0\\ L\leq v\leq U \end{array} \end{array}$$

where *v* is the vector of fluxes of the reactions, *c* is a vector of weights indicating the contribution of the flux of each reaction to the objective function, S is the stoichiometric matrix, and *L* and *U* are lower and upper flux bounds. The maximization of the objective function, $c^{T}\cdot v$, yields an optimal distribution of fluxes that satisfies the steady-state condition, S·v=0, and the flux bounds, L≤v≤U.

FVA builds upon FBA and determines the range of fluxes of each reaction that is compatible with a given fraction *q* of the optimal value of the objective function in (3). FVA provides insight into the flexibility of metabolic pathways under different conditions, and can be computed as follows:

(4) $$\begin{array}{c} \begin{array}{c} \mathrm{Max}/\mathrm{Min}\;v(r)\\ S\cdot v=0\\ L\leq v\leq U\\ c^{T}\cdot v\geq q\cdot \mu \end{array} \end{array}$$

where v(r) denotes the flux of reaction *r*, μ is the solution of (3), and q∈[0,1] sets the fraction of the optimal value μ that is required (*q* is usually very close to 1). If v(r) in Equation (4) is maximized (minimized), then the maximum (minimum) steady-state flux of reaction *r* that is compatible with the optimal value of $c^{T}\cdot v$ is computed.

FBA and FVA were used to elucidate the behavior of the enhanced constraint-based model. For the simulations, we used the value of glucose uptake in the kinetic model (0.23 $mMs^{-1}$) as the default constraint for the constraint-based model. The biomass reaction, *BIOMASS_Ec_iML1515_WT_75p37M*, was set as the objective function.

### 2.5 Technical analysis of the simulations

Regarding the kinetic model simulations, we used libRoadRunner 2.7.0 which is a C/C++ library that supports simulation of SBML-based models (Welsh *et al.* 2023). The computation of the kinetic bounds takes approximately 3 seconds. The kinetic simulations reproduced an M9 minimal medium with glucose as sole carbon source, with a dilution rate of 0.1 h^−1^, 37°C, pH = 7.0, pO_2_  ≥ 20%.

Regarding the genome-scale model, we retrieved the original iML1515 model from the BiGG Models database (King *et al.* 2016). COBRApy (Ebrahim *et al.* 2013), a Python package to reconstruct constraint-based models, was used to translate the kinetic bounds and to run the FBA and FVA analyses. Loading the model, mapping and translating the kinetic bounds, and running the FBA and FVA analysis for the 29 mapped reactions take less than one minute.

All simulations were performed on a workstation featuring an 11th Gen Intel® Core™ i7-1165G7 processor (2.80 GHz, 4 cores) and 15 GB of RAM, running Ubuntu 22.04.3 with Linux kernel version 6.8.0.

## 3 Results

### 3.1 Translating fluxes between the kinetic and constraint-based models

#### 3.1.1 Mapping of reactions

As part of translating fluxes from the kinetic model to the constraint-based model, reactions in the kinetic model were mapped to their equivalents in the constraint-based model (File 1, available as supplementary data at *Bioinformatics Advances* online). As it will be shown, even if the metabolic network of the constraint-based model is larger than that of the kinetic model, a one-to-one mapping of the set of reactions of the kinetic model onto the set of reactions of the constraint-based model does not always exist.

Some reactions of the kinetic model are identical to those of the constraint-based model, i.e. the sets of reactants, products, and the stoichiometry are the same. In such a case, it suffices to convert the flux units to translate the flux bounds of the kinetic model to the constrained-based model, see (5).

The unit conversion factor *c* is necessary because the kinetic and constraint-based models use different units for fluxes: the kinetic model expresses fluxes in $mM\;s^{-1}$, while the constraint-based model uses $\mathrm{mmol}\;g_{DW}^{-1}\;h^{-1}$. To convert between these units, we used a cell volume of $1.77\cdot 10^{-3}\;L\;g_{DW}^{-1}$, as reported in Millard *et al.* (2017). Specifically, a flux $f(r)\;mM\;s^{-1}$ of a reaction *r* in the kinetic model corresponds to:

$$\underset{\text{flux of r in the kinetics imulation}}{\underset{︸}{\left(f(r)\;mM\;s^{-1}\right)}}\cdot \underset{\text{cell volume}}{\underset{︸}{\left(1.77\cdot 10^{-3}\;L\;g_{DW}^{-1}\right)}}\cdot (3600\;s\;h^{-1})$$

 

(5) $$=6.372\;\cdot f(r)\;\mathrm{mmol}\;g_{DW}^{-1}\;h^{-1}$$

i.e., the conversion factor *c* is 6.372. The value f(r) will be the basis for the computation of the kinetic bounds detailed in *Translation of fluxes*.

However, non-identical reactions cannot be translated in the same way. All the equivalent reactions between models were classified in the following five categories, which were used to define equations that translate flux bounds to the constraint-based model (see Table 1 for an example reaction of each category):

**Table 1.** Categories of reactions to map fluxes from the kinetic to the constraint-based model.

Category	Reaction in kinetic model	Reaction in constraint-based model
*C*1	FDP→F6P+P	$\mathrm{FDP}+H_{2}O\to F6P+P$
*C*2	$\mathrm{MAL}+\mathrm{NAD}\to \mathrm{NADH}+\mathrm{PYR}+HCO_{3}^{-}$	$\mathrm{MAL}+\mathrm{NAD}\to \mathrm{NADH}+\mathrm{PYR}+CO_{2}$
*C*3	ICIT↔GLX+SUC	ICIT→GLX+SUC
*C*4	ADP+BPG↔ATP+PGA3	PGA3+ATP↔BPG+ADP
*C*5	Subnetworks with different structure (no one-to-one mapping)

1. **Difference in a small chemical species**: Reactions that differ in the species $H^{+}$ or $H_{2}O$.
2. **Acid hydrolysis**: Reactions in which the only difference is that $CO_{2}$ is used in the constraint-based model instead of $HCO_{3}^{-}$, which is used in the kinetic model.
3. **Different reversibility**: Reactions that are reversible in one model and irreversible in the other model.
4. **Reversed**: Reactions that are reversed in the models, i.e. the metabolites that are reactants in one model are products in the other model.
5. **Subnetworks with different structure**: This category includes sets of reactions that perform the same chemical function but differ in the number of metabolites and in the connection pattern; thus, a one-to-one mapping is not possible.

#### 3.1.2 Translation of fluxes

To translate the fluxes, we used the fluxes obtained by the kinetic simulations, f(r), as the initial input and proceeded as follows: First, we converted the units between models by multiplying f(r) by *c*. Next, we introduced different levels of uncertainty to the translated fluxes so that they fell within the interval [L(r),U(r)], where L(r), resp. U(r), is obtained by scaling the flux by (1−d), resp. (1+d). Thus, the computation of lower and upper flux bounds, L(r) and U(r), for a given reaction *r* of the constraint-based model from the steady-state fluxes of the kinetic model was derived by using Equations (6):

(6) $$\begin{array}{c} L(r)=c(1-d)\cdot f(r)\\ U(r)=c(1+d)\cdot f(r) \end{array}$$

where:

-
f(r) is the flux of reaction *r* obtained from the simulation of the kinetic model. Note that all the computed fluxes of the kinetic model are positive (Table 1, available as supplementary data at *Bioinformatics Advances* online).
-
d > 0 is a real parameter that accounts for the level of uncertainty of the model.
- *c* is the unit conversion factor.
-
L(r) is the lower flux bound of reaction *r*.
-
U(r) is the upper flux bound of reaction *r*.

The flux from the kinetic model, f(r), serves as the central estimate for the flux in the constraint-based model; *d* represents the relative uncertainty of the kinetic flux; and *c* is the unit conversion factor. For example, if d=0.1 (i.e. uncertainty of 10%), then the lower bound of reaction *r* in the constraint-based model will be 0.9 times the flux of the reaction in the kinetic model, and the upper bound of the reaction in the constraint-based model will be 1.1 times the flux of the reaction in the kinetic model. Equation (6) effectively applies a proportional uncertainty to $V_{\max}$, which is proportional to the enzyme abundance and the catalytic constant. Such a proportional uncertainty reflects fold-changes to enzyme expression which is usual for biological systems. Accounting for intervals of fluxes can be biologically interpreted as simulating perturbations in parameters that influence flux, such as enzyme abundance and the reaction’s catalytic constant [Equation (1)].

In general, the kinetic bounds L(r) and U(r) obtained by applying Equation (6) were more restrictive than the original default bounds of the genome-scale model iML1515.

For the reactions in categories *C*1, *C*2, and *C*3, the flux bounds of the kinetic model can be applied directly to the constraint-based model after unit conversion, i.e. as in Equation (6). This is because there is no discrepancy in the direction of the reaction or in the connectivity of the metabolites and reactions between models.

In category *C*4, reactants and products are swapped, i.e. the directionality of reactants and products is inverted between models, and hence, flux bounds of the constraint-based model must also be inverted:

(7) $$\begin{array}{c} L(r)=-c(1+d)\cdot f(r)\\ U(r)=-c(1-d)\cdot f(r) \end{array}$$

A procedure to compute flux bounds for reactions in category *C*5 is introduced in the Supplemental Text, available as supplementary data at *Bioinformatics Advances* online section (see the Supplemental Material, available as supplementary data at *Bioinformatics Advances* online).

### 3.2 Enriching the constraint-based model predicts more realistic flux properties

To assess how changes in particular reactions can affect the set of optimal fluxes over the reactions of a genome-scale metabolic model, we sequentially restricted the flux bounds of the constraint-based model. Specifically, we sequentially added the bounds of 29 reactions following the glycolysis pathway (Table 1, available as supplementary data at *Bioinformatics Advances* online), following Equation (6). We chose these reactions because we used a glucose-limited metabolic model and because adding constraints to more reactions causes the model to become infeasible as it overly restricts the linear programming solution space for FBA. After each reaction was added, we performed FBA and FVA, setting the parameter for the fraction of optimum as q=0.999 for FVA—this is relevant in practice because constraining the solution to the edge of the feasible state, i.e. q=1, might lead to a reduced solution space which may result in a failure during the optimization process (Loghmani *et al.* 2022).

This approach allowed us to track specific changes in the behavior of the model as we implemented the kinetic bounds of each reaction. In particular, the solutions of FBA and FVA obtained after implementing flux bounds on each reaction in Table 1, available as supplementary data at *Bioinformatics Advances* online were used to: (i) estimate the growth rate; (ii) estimate the feasible range FV(r) of fluxes of reactions before and after enriching the constraint-based model with the kinetic bounds; and (iii) compute the number of *dormant* reactions. More precisely, FV(r) represents the potential flux variability of reaction *r* and is defined as follows:

(8) FV(r)=maxF(r)−minF(r)

where minF(r) and maxF(r) are the minimum and maximum fluxes of reaction *r* computed by FVA.

On the other hand, a reaction *r* is said to be *dormant* when its steady-state flux is necessarily zero, i.e.:

(9) |minF(r)|≤ϵ   and   |maxF(r)|≤ϵ

where ϵ is a small real quantity that accounts for the solver accuracy. To avoid numerical issues produced by the solver accuracy, ϵ has been set to $10^{-2}$  $\mathrm{mmol}\;g_{DW}^{-1}\;h^{-1}$ in our experiments.

#### 3.2.1 Kinetic bound uncertainty of 1%

**Figure 2.**
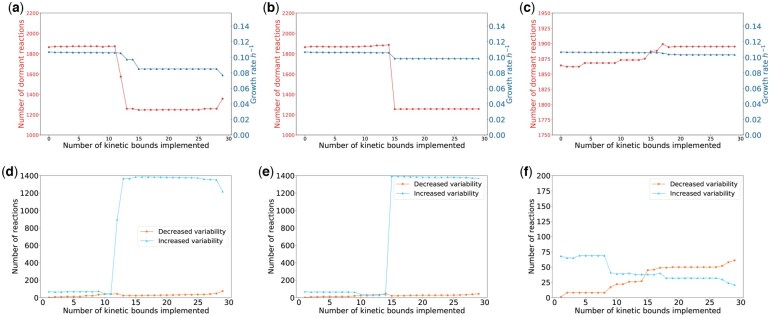
(a) Growth rate (blue line, triangle points) and number of dormant reactions (red line, round points) with uncertainty parameter d=0.01 as the number of implemented kinetic bounds increases, (b) with uncertainty parameter d=0.06 and (c) with uncertainty parameter d=0.1. (d) Number of reactions with more variability (blue line, triangle points) and number of reactions with less variability (orange line, round points) with respect to the original model as the number of implemented kinetic bounds increases with *d *= 0.01, (e) with *d *= 0.06, and (f) with *d *= 0.1.

To assess the effects of kinetic bound uncertainty values on the behavior of the model, we imposed uncertainty values *d* of 0.01, 0.06, and 0.1 to capture a large range of model behaviors, ranging from the lowest level of uncertainty that results in a feasible solution to behaviors similar to the original model. To assess whether imposing a kinetic bound with uncertainty d=0.01 makes the model more realistic, we computed the growth rate, the variability of each reaction, and the number of dormant reactions as each bound was added to the constraint-based model. Figure 2a shows that as bounds were added, the growth rate was either maintained or decreased. This observation is expected because kinetic bounds involve a more constrained model.

The number of dormant reactions is mostly either maintained or decreased as the kinetic bounds are sequentially implemented. Small changes in the growth rate affect the number of dormant reactions. When reaction number 12 is added, the number of dormant reactions falls drastically, i.e. some reactions were dormant, but are awakened once the kinetic bounds are applied. This change is due to the combined effect of the implemented kinetic bounds and not to the addition of the bounds of a particular reaction. This combination of kinetic bounds leads to a change in the FBA solution, which was characterized by a reallocation of the optimal fluxes in order to find another optimal solution. In contrast, the original model without kinetic bounds is too loose and the optimization of the growth rate implies that most of the reactions are dormant or inactive, which is not realistic. Our simulations, hence, imply that the addition of kinetic bounds results in a more realistic set of optimal fluxes with fewer dormant reactions and a lower growth rate.

In order to assess the flux variability over reactions, FV(r) was used, see Equation (8). In particular, we computed the number of reactions whose FV(r) increased and the number of reactions whose FV(r) decreased after the addition of kinetic bounds with respect to the original constraint-based model. Figure 2d reports the number of reactions whose FV(r) increases and the number of reactions whose FV(r) decreases. Figure 2d is complementary to Fig. 2a and shows that when the number of dormant reactions falls, several reactions increase their FV(r). This observation suggests that many of the dormant reactions increased their FV(r), so that metabolic flux is available through these reactions.

These results suggested that when the kinetic bounds of the reactions in Table 1, available as supplementary data at *Bioinformatics Advances* online are added to the constraint-based model, the fluxes tend to reallocate in order to find a feasible solution. The limitations introduced by these new constraints do not allow flux values to achieve the same set of optimal fluxes as when the model has no flux bounds determined by the kinetic model. This leads to the following hypothesis: performing FBA on a model that has very loose constraints finds optimal solutions where many reactions in the model are dormant. Nevertheless, if we introduce additional constraints such as the kinetic bounds, the freedom of fluxes of certain reactions is reduced. Reactions that originally could get a wide range of fluxes, can no longer do so when they are constrained by the kinetic bounds. Thus, the original flux of a reaction has to reallocate through other reactions that were inactive. As a result, the optimal solution decreases and the optimal pathways that lead to the original solution are bounded, so new reactions have to activate and fluxes must be reallocated over the network to find a new optimal solution.

#### 3.2.2 Kinetic bound uncertainty of 6%

When we set an uncertainty of d=0.06, the number of dormant reactions and flux variability followed a similar trend to the one displayed with an uncertainty of d=0.03, Fig. 2a and d. Nevertheless, in this case, the number of dormant reactions fell and the growth rate changed substantially when the kinetic bounds of reaction number 15 were added to the constraint-based model, see Fig. 2b. Figure 2e shows that the change in the set of optimal fluxes occurs when the bounds of the reaction number 15 are implemented (the number of reactions that increase their FV(r) rises radically).

#### 3.2.3 Kinetic bound uncertainty of 10%

Finally, when the level of uncertainty was set to 10% (*d *= 0.1), neither the growth rate nor the dormant reactions are substantially affected, see Figs. 2c and f.

In general, when the uncertainty is low, the number of dormant reactions falls after the addition of fewer kinetic bounds, leading to an earlier rise in reactions with increased variability. This is expected because a lower uncertainty in the kinetic bounds means that the model is more constrained, so the reallocation of metabolic fluxes happens earlier. This also results in reaching a lower optimal solution, i.e, growth rate. In addition, higher uncertainty in the kinetic bounds implies that more kinetic bounds can be implemented.

**Figure 3.**
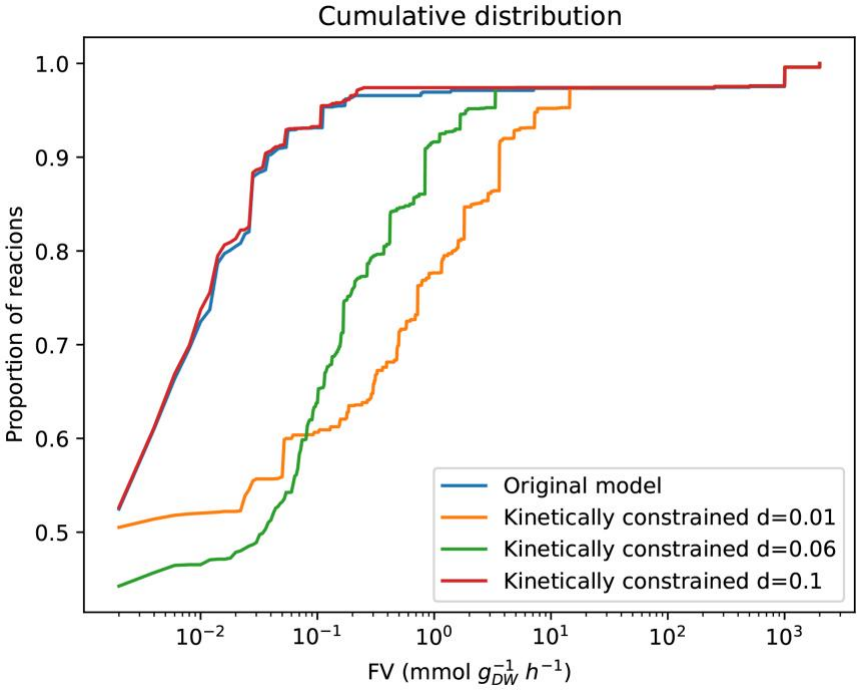
Cumulative distribution of *FV(r)* values in the original model (blue curve) and in the constraint-based model after the kinetic bounds for all reactions were added (kinetically constrained model) with *d *= 0.01 (orange curve), *d *= 0.06 (green curve), and *d *= 0.1 (red curve).

Additionally, to examine the variability of the reaction bounds, we computed the distribution of FV(r) values across reactions for the three uncertainty levels considered, d=0.01, d=0.06, and d=0.1. The cumulative distribution of the FV(r) in Fig. 3 shows that more than 90% of the total reactions had an FV(r) value lower than $10^{-1}$  $\mathrm{mmol}\;g_{DW}^{-1}\;h^{-1}$ when FVA was performed on the original model and in the model enriched with kinetic bounds with an uncertainty of d=0.1. In contrast, less than 70% of reactions had an *FV* value lower than $10^{-1}$  $\mathrm{mmol}\;g_{DW}^{-1}\;h^{-1}$ when FVA was performed on the kinetically constrained model under d=0.06 and d=0.01 uncertainty values. As *d* increases, the percentage of reactions for a given *FV* is higher. This means that higher levels of uncertainty in the translation of the kinetic bounds results in a lower *FV*. This agrees with the aforementioned finding: new reactions have to activate and fluxes must reallocate through the network to find a new optimal solution when kinetic constraints are added. Thus, a low uncertainty implies that more reactions increase their flux variability. For a more detailed description of how the levels of uncertainty affect the behavior of the model, see Supplemental Material: *A finer sampling on the uncertainty: Original Model*, available as supplementary data at *Bioinformatics Advances* online.

### 3.3 Enriched constraint-based model to simulate citramalate production

#### 3.3.1 Inclusion of citramalate production in the constraint-based model

**Figure 4.**
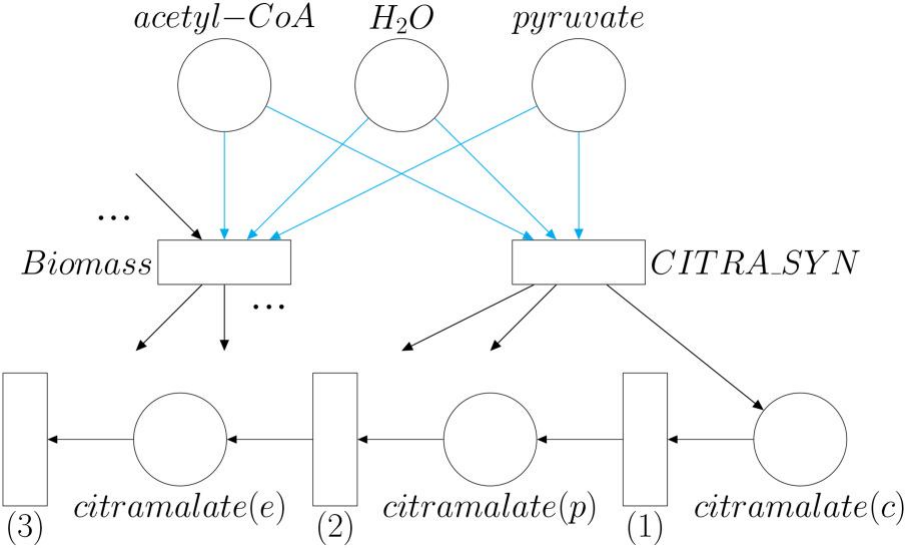
Simplified Petri net depicting the location of bifurcation (cyan arrows) between growth and citramalate production. Cyan arrows indicate where the bifurcation of fluxes occurs. Reactions with numbers (1), (2), and (3) represent the reactions *CitraTransp1*, *CitraTransp2*, and *EX_Citramalate*, respectively, added to the constraint-based model so that citramalate can leave the system. For simplicity, some places were not represented so their corresponding arrows point no element. The “…” means that more metabolites are involved in the reaction where they appear: if “…” appears next to an incoming arc, there are more reactants in that reaction; if “…” is next to an outgoing arc, more products take part in that reaction although not depicted.

One of the main motivations in this paper is to solve the problem that arises when there are two competing objective functions in the model. In a realistic model, there would be a trade-off between the growth rate and the production of citramalate. However, depending on the objective function set, a straightforward application of FBA results in the carbon source being fully directed either to cellular growth or to citramalate production (Fig. 4). The kinetic model does not suffer from this problem, since its differential equations impose positive fluxes in the reactions involved in the split of the pathways between growth and product formation as long as the concentration of the reactants is positive. Thus, to make the constraint-based model more realistic, we forced its growth rate to be in an interval that depends on the growth rate generated by the kinetic model and the uncertainty *d*, i.e. we added a new constraint specifying that the flux of the biomass reaction of iML1515 must be in the interval $\left[\mu_{k}(1-d),\mu_{k}(1+d)\right]$, where $\mu_{k}$ is the growth rate generated by the kinetic model. This guarantees a strictly positive growth rate even if the objective function is the maximization of citramalate production.

We modified the original constraint-based model of *E. coli* to model the synthesis and secretion of citramalate by the cell. The main reaction that was added to the model is (2) whose ID in the model is *CIMA*. Since the *E. coli* model has three compartments (cytosol, periplasm, and extracellular), reactions that model the transport of citramalate from the cytosol to the periplasm (ID *CitraTransp1*) and from the periplasm to the extracellular compartment (ID *CitraTransp2*) were included as well. Finally, an exchange reaction (ID *EX_Citramalate*) that removes the citramalate from the extracellular compartment was added. This exchange reaction prevents the accumulation of extracellular citramalate and allows a steady state to be reached (Fig. 4). Subsequently, we chose this exchange reaction as the objective function in the FBA simulations.

#### 3.3.2 Inclusion of citramalate production in the kinetic model

To model citramalate synthesis using the kinetic model, we added a new reaction, *CITRA_SYN*, to the model. The stoichiometry of this reaction is given by (2) and it follows the Michaelis–Menten kinetic law (10):

(10) $$\frac{d[\mathrm{citramalate}]}{dt}=\frac{V_{\max}\cdot [\mathrm{acetyl}-\mathrm{CoA}]}{[\mathrm{acetyl}-\mathrm{CoA}]+K_{m}}$$

which assumes that pyruvate is saturating (Takamura and Nomura 1988, Atsumi and Liao 2008, Mitsui *et al.* 2024). The $K_{m}$ was set to 0.495 mM (Webb *et al.* 2018), and the $V_{\max}$ was calculated from the maximum activity of *CimA3.7* and the cell volume. More precisely, since the maximum activity of *CimA3.7* in one cell is $24.34\cdot 10^{-10}n\mathrm{mol}/s$ (Webb *et al.* 2018), and that the cell volume is approximately $6\cdot 10^{-16}L$ (Kubitschek 1990), the $V_{\max}$ of *CimA3.7* can be calculated as follows: $V_{\max}=\frac{24.34\cdot 10^{-10}\;\mathrm{nmol}/s}{6\cdot 10^{-16}\;L}=\frac{24.34}{6}\cdot 10^{6}\;\mathrm{nmol}/L/s=4.06\;mM/s$.

#### 3.3.3 Effect of varying kinetic bounds in the genome-scale model

**Figure 5.**
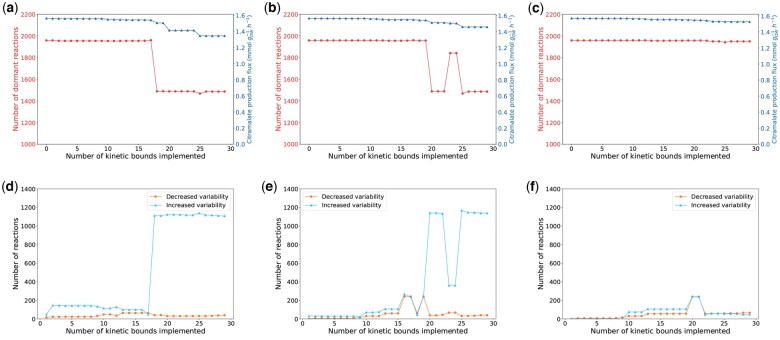
(a) Dormant reactions (red line, round points) and citramalate production flux (blue line, triangle points) in the citramalate-producing model as the number of implemented kinetic bounds increases when d=0.1, (b) when d=0.3, and (c) when d=0.5. Number of reactions with more variability (blue line, triangle points) and number of reactions with less variability (orange line, round points) in the citramalate-producing model with respect to the original model as the number of implemented kinetic bounds increases for (d) d=0.1, (e) d=0.3, and (f) d=0.5.

The constraint-based model including the citramalate synthesis reaction was enriched with kinetic information in the same way as the wild-type constraint-based model. However, we used different uncertainty levels for this model (d=0.1, d=0.3, d=0.5) based on the observation of changes in the previously analyzed metrics, i.e. the number of dormant reactions or the number of reactions whose variability drastically varies. Figure 5a–c report the number of dormant reactions and the maximized citramalate production flux after implementing kinetic bounds. They all show three different states of the citramalate-producing model as a function of the uncertainty level: Fig. 5a shows a pronounced fall in the number of dormant reactions, which coincides with the first substantial reduction in the citramalate production flux; Fig. 5b shows a similar behavior but there is an intermediate state (when 23 kinetic bounds are implemented) where many activated reactions deactivate. Eventually, the number of dormant reactions falls almost to the same value as when d=0.1; Fig. 5c represents a state where implementing kinetic bounds with a level of uncertainty d=0.5 has little effect on the number of dormant reactions.

Notice that the kinetic bounds have been implemented following the order of glucose metabolism, see Table 1, available as supplementary data at *Bioinformatics Advances* online. Section *On the sequence of kinetic bounds implemented* in the Supplemental Material, available as supplementary data at *Bioinformatics Advances* online addresses the effect of a different implementation order on the results.

Figure 5d–f show the number of reactions whose flux variability, FV(r), increases (blue curve) and decreases (red curve) after including the kinetic bounds with a level of uncertainty of 10%, 30%, and 50% respectively. They show the complementarity between the number of dormant reactions and the number of reactions that increase their variability after implementing the kinetic bounds, i.e. when the number of dormant reactions falls, the number of reactions that increase their variability augments since many of the former dormant reactions start to carry a non-negative flux, increasing their flux variability. A more detailed description of how the levels of uncertainty affect the behavior of the model can be found in Supplemental Material: *A finer sampling on the uncertainty: Citramalate-producing model*, available as supplementary data at *Bioinformatics Advances* online.

**Figure 6.**
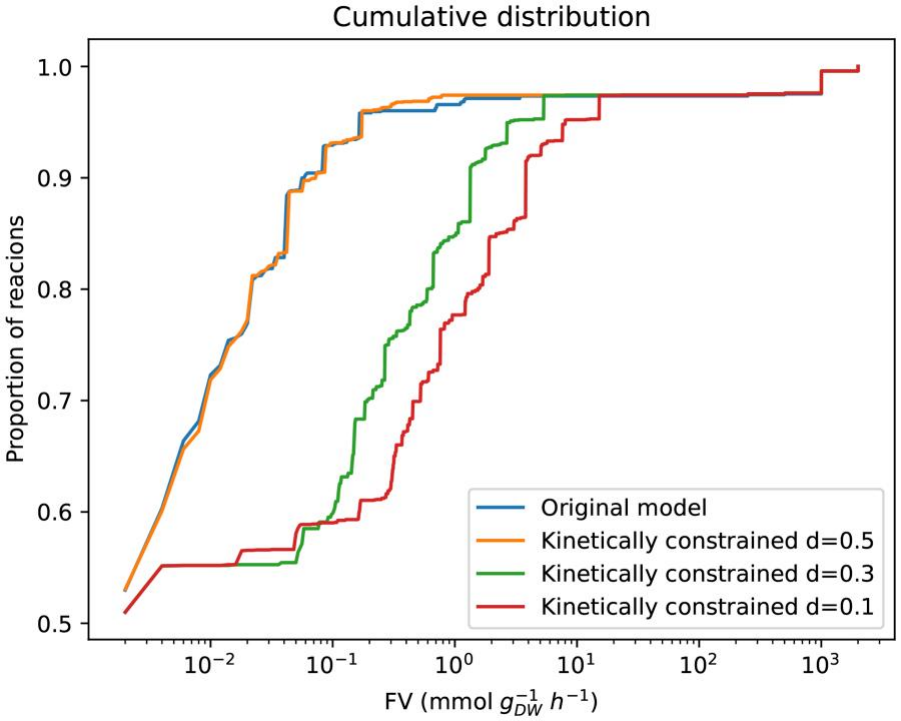
Cumulative distribution of *FV(r)* values in the enriched original model (blue curve) and in the constraint-based model after the kinetic bounds for all reactions were added (kinetically constrained model) with *d *= 0.1 (red curve), *d *= 0.3 (green curve), and *d *= 0.5 (orange curve).

Once again, to assess the variability of the reaction bounds, we calculated the distribution of FV(r) values across reactions. Figure 6 shows that lower levels of uncertainty imply more reactions with higher variability, which is in agreement with Fig. 3. In summary, lower levels of uncertainty imply more reactions with higher variability.

#### 3.3.4 Validation of citramalate efficiency in the kinetically constrained model

To validate the kinetically constrained model, we compared the predictions of the model with the experimental values reported in Webb *et al.* (2018). In our simulations, the flux of citramalate, and hence the conversion efficiency, depends on the uncertainty used to implement the kinetic bounds in the model, see Table 2.

**Table 2.** Conversion efficiencies from glucose to citramalate computed under different conditions.^a^

Conversion efficiency (g/g)				
Experimental	Original model	Uncertainty *d *= 0.5	Uncertainty *d *= 0.3	Uncertainty *d *= 0.1
0.48 ± 0.03	0.95	0.857	0.812	0.755

a *Experimental* corresponds to the experimental value for the conversion efficiency in *E. coli* according to (Webb *et al.* 2018) where the flow rate was manually adjusted as needed to sustain a pseudo-exponential growth rate of about 0.25 $h^{-1}$ and to prevent excess glucose accumulation in the culture; *Original* is the conversion efficiency in the computational model iML1515 without additional constraints performing FBA; and the rest of the conditions refer to the enriched computational model with kinetic bounds integrated with different levels of uncertainty (10%, 6%, and 3%).

Glucose conversion to citramalate is highest in the original model without kinetic bounds, likely overestimating efficiency due to too loose constraints. This also implies no growth when maximizing citramalate flux which is unrealistic. Adding kinetic bounds reduced the solution value and solved the no-growth problem, aligning efficiency closer to experimental results, with further decreases as uncertainty diminished.

## 4 Discussion

As expected, the glucose conversion into citramalate is highest for the original model, i.e. without kinetic bounds translated to the constraint-based model. This value likely overestimates the efficiency due to the lack of realistic constraints in the model. Thus, implementing the kinetic bounds into the constraint-based model decreased the value of the solution and brought the conversion efficiency closer to the experimental one. The efficiency progressively decreased as the level of uncertainty was reduced. However, there is still some discrepancy between the experimental value in the ones predicted by our enhanced model, probably because our model overlooks other constraints such as thermodynamic, regulatory, and enzyme abundance constraints that may help limit the solution to an even more realistic citramalate production. Although constructing constraint-based models is straightforward, traditional constraint-based models are imprecise and unreliable because they lack kinetic and proteomic information. Nevertheless, advances in omics enabled the extension of these models to incorporate single-cell RNA-seq, proteomic and transcriptomic data (Tian and Reed 2018, Damiani *et al.* 2019, Alghamdi *et al.* 2021). Stoichiometry and flux bounds of reactions are the only constraints of traditional constraint-based models. The default flux bounds assigned to most reactions are very loose and fail to realistically constrain the fluxes of reactions.

Therefore, we aimed to make more realistic constraint-based genome-scale models by combining the properties of the former and kinetic models. Here, we evaluated a genome-scale model of *E. coli* that we enriched with the data obtained from a simulation of the steady state of a kinetic model. The kinetic model is smaller than the constraint-based model and holds the kinetic information for the reactions involved in the carbon metabolism. We also created a new model that can produce citramalate based on kinetic parameters that were previously determined experimentally.

A key challenge in reconstructed networks is the occurrence of dormant reactions, which are constrained to zero flux and therefore remain inactive under steady-state conditions. The reduction of the number of non-functional reactions has been used to validate models as they provide better coverage of the space of metabolic reactions and exhibit fewer dormant reactions (and hence, considerably more reactions that can carry a flux different from zero) (Burgard *et al.* 2004, Chindelevitch *et al.* 2012).

We demonstrated that the enriched model, with or without citramalate synthesis, is more realistic because adding kinetic bounds reduces dormant reactions and increases reaction flux variability. With kinetic constraints, flux distributions require activating alternative, less efficient pathways, reflecting real-world limitations absent in unconstrained models where flux is unrestricted. Furthermore, we demonstrated that increased uncertainty of kinetic bounds leads to a model that behaves more closely to the unconstrained model. In contrast to other studies such as Massaiu *et al.* (2019) and Yeo *et al.* (2020), in some cases, adding kinetic constraints to a genome-scale model can increase the flexibility of reaction fluxes (Cotten and Reed 2013). This effect arises when kinetic constraints unblock alternative pathways or allow previously restricted reactions to carry flux, effectively expanding the variability of the metabolic fluxes. Rather than strictly reducing variability, kinetic constraints can sometimes enable a redistribution of fluxes that activates additional metabolic routes under specific conditions.

In the extended citramalate-producing constraint-based model, a bifurcation channels nutrient flux either towards biomass or citramalate production. We resolved this by fixing the growth rate to the value from the kinetic model with the citramalate synthesis reaction added. Implementing kinetic bounds with uncertainty improved the model’s prediction of glucose-to-citramalate conversion efficiency, aligning it with experimental data. Our approach, while demonstrated using *E. coli*, is designed to be applicable to other organisms and metabolic networks, even those with limited kinetic data, by allowing flexible mapping between kinetic and constraint-based models. Despite relying on kinetic parameters, we show that significant predictive improvements can be achieved with only partial data—just 29 of 2712 reactions in the iML1515 model were constrained. Advances in machine learning now enable the estimation of kinetic parameters under certain conditions, as implemented in tools like GECKO 3.0 (with DLKcat) (Domenzain *et al.* 2022), RENAISSANCE (Choudhury *et al.* 2024), and REKINDLE (Choudhury *et al.* 2022). Using the large-scale iML1515 model, we demonstrate that even limited kinetic coverage can effectively reduce the solution space, with constraints on central metabolism reactions indirectly influencing the broader network due to their high connectivity. While constraining a constraint-based model using kinetic model outputs enhances realism, this approach inherits any inaccuracies from the kinetic model. The enhanced model realistically captures the citramalate production and growth rate trade-off and reflects resource allocation. It thus approximates predictions of growth and citramalate productivity in *E. coli* to a more realistic scenario.

## Supplementary Material

vbaf166_Supplementary_Data
