# Supplementary material for: Enhancing genome-scale metabolic models with kinetic data: resolving growth and citramalate production trade-offs in Escherichia coli
Source: Bioinform Adv. 2025 Jul 12;5(1):vbaf166. doi: 10.1093/bioadv/vbaf166 (PMC12341681; doi:10.1093/bioadv/vbaf166)
Supplement: vbaf166_Supplementary_Data [file vbaf166_supplementary_data.zip › BioAd_supplementary.pdf]

SUPPLEMENTAL MATERIAL

Supplemental Text

Subnetworks with different structure

Because a one-to-one mapping is not possible for reactions in category $C5$, the flux bounds of the kinetic model cannot be applied directly to the constraint-based model.The three subnetworks in the kinetic model that belong to category $C5$ have different structures and, hence, will be considered separately. In the following graphical representations, a Petri net transition depicted as a double rectangle models a reversible reaction.

Subnetwork 1. The transformation of periplasmic glucose ($GLPc$) and phosphoenolpyruvate (PEP) into glucose-6-phosphate ($G6P$) and pyruvate (PYR) is modeled by 5 reactions, *PTS_0*, *PTS_1*, *PTS_2*, *PTS_3* and *PTS_4*, in the kinetic model, see Figure 1, and by one reaction, $GLCptspp$, in the constraint-based model, see Figure 2.

In the steady state, the overall incoming flux to a metabolite must be equal to its overall outgoing flux. Thus, the following equation will hold in the kinetic model:

$$f(PTS\_0) = f(PTS\_1) = f(PTS\_2) = f(PTS\_3) = f(PTS\_4) \tag{1}$$

where $[E]$ is the enzyme concentration and k_{cat} is the turnover number.

Hence, the flux of any reaction in Figure 1 can be taken to bound reaction $GLCptspp$ in the constraint-based model. From Equation 6 (Main Text), appropriate lower and upper flux bounds for reaction $GLCptspp$ can be computed by:

$$\begin{aligned} L(GLCptspp) &= (1-d) \cdot c \cdot f(PTS\_0) \\ U(GLCptspp) &= (1+d) \cdot c \cdot f(PTS\_0) \end{aligned} \tag{2}$$

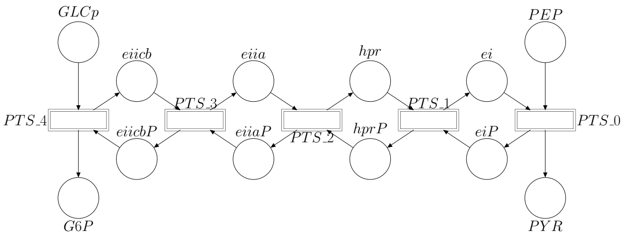

Fig. 1. Petri net representation of the **Subnetwork 1** in the kinetic model, where the reactions are PTS_0, PTS_1, PTS_2, PTS_3 and PTS_4, the reactants are periplasmic glucose ($GLCp$) and phosphoenolpyruvate (PEP), and the products are glucose-6-phosphate ($G6P$) and pyruvate (PYR). The rest of the places represent intermediates participating in the reactions.

Subnetwork 2. The transformation of ubiquinone-8 (Q) and succinate (SUC) into fumarate (FUM) and ubiquinol-8 ($QH2$) is modeled by two reactions (SDH and SQR) in the kinetic model, see Figure 3, and by one reaction ($SUCDi$) in the constraint-based model, see Figure 4. Following the reasoning for *Subnetwork 1*, the two reactions of the kinetic model must have the same flux in the steady state. Thus, it holds:

$$f(SDH) = f(SQR) \tag{3}$$

and the following bounds for the reaction $SUCDi$ in the constraint-based model are obtained:

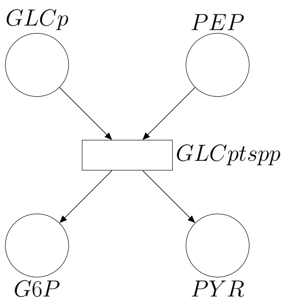

Fig. 2. Petri net representation of the **Subnetwork 1** in the constraint-based model, where $GLCptspp$ is the only reaction (notice that this reaction corresponds with the reactions in Figure 1 of the kinetic model). The reactants of the reactions are periplasmic glucose ($GLCp$) and phosphoenolpyruvate (PEP), the products are glucose-6-phosphate ($G6P$) and pyruvate (PYR).

$$\begin{aligned} L(SUCDi) &= (1-d) \cdot c \cdot f(SDH) \\ U(SUCDi) &= (1+d) \cdot c \cdot f(SDH) \end{aligned} \tag{4}$$

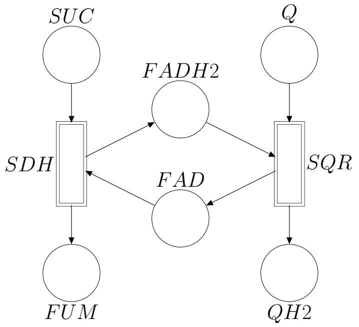

Fig. 3. Petri net representation of the **Subnetwork 2** in the kinetic model.

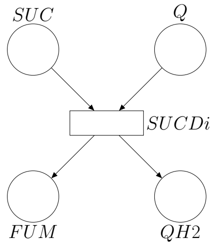

Fig. 4. Petri net representation of the **Subnetwork 2** in the constraint-based model.

Subnetwork 3. The pentose phosphate pathway (PPP) is modeled differently in the kinetic and the constraint-based model. Namely, it is modeled by reactions *X5P_GAP_TKT*, *S7P_R5P_TKT*, *F6P_GAP_TAL*, *S7P_E4P_TAL* and *F6P_E4P_TKT* in the kinetic model, see Figure 5; and by $TKT1$, $TKT2$ and $TALA$ in the constraint-based model, see Figure 6. To map flux bounds in these subnetworks, we observe that various reactions in both models are equivalent, namely:

$$\underbrace{S7P\_R5P\_TKT + X5P\_GAP\_TKT}_{\text{kinetic}} = \underbrace{TKT1}_{\text{constraint-based}} \quad (5a)$$

$$\underbrace{F6P\_E4P\_TKT + X5P\_GAP\_TKT}_{\text{kinetic}} = \underbrace{TKT2}_{\text{constraint-based}} \quad (5b)$$

$$\underbrace{F6P\_GAP\_TAL + S7P\_E4P\_TAL}_{\text{kinetic}} = \underbrace{TALA}_{\text{constraint-based}} \quad (5c)$$

These equivalences are obtained by taking into account the cumulative effect of reactions. For instance, Equation (5b) is obtained by:

$$\begin{array}{l} \underbrace{F6P\_E4P\_TKT}_{\text{reaction ID}} : \underbrace{E4P + \cancel{tktC2}}_{\text{reactants}} \rightarrow \underbrace{F6P + \cancel{tkt}}_{\text{products}} \\ \underbrace{X5P\_GAP\_TKT}_{\text{reaction ID}} : \underbrace{\cancel{tkt} + X5P}_{\text{reactants}} \rightarrow \underbrace{GAP + \cancel{tktC2}}_{\text{products}} \\ \hline \underbrace{TKT2}_{\text{reaction ID}} : \underbrace{E4P + X5P \leftrightarrow F6P + GAP}_{\text{reaction}} \end{array} \quad (6)$$

The sum of two reactions in the kinetic model, (*F6P_E4P_TKT* and *X5P_GAP_TKT*) results in reaction *TKT2* from the constraint-based model. This means that the kinetic model uses two reactions to produce metabolites *F6P* and *GAP* when *E4P* and *X5P* react. Two reaction intermediates are also specified (*tkt* and *tktC2*). In contrast, the constraint-based model ignores these intermediates and uses only one reaction to catalyze the same process. Similar differences also apply for the Equations (5a) and (5c).

To determine flux bounds, consider that under the steady state assumption, metabolite concentrations are assumed to be constant, i.e. the incoming flux to a metabolite must be equal to its outgoing flux. This assumption allows us to establish relationships among the steady-state fluxes of reactions in the network depicted in Figure 5. In particular, the relationship in (7) can be deduced by assuming that the concentration of *tktC2* and *tkt* is constant:

$$f(X5P\_GAP\_TKT) = f(S7P\_R5P\_TKT) + f(F6P\_E4P\_TKT) \quad (7)$$

Similarly, equation (8) is obtained by considering metabolites *tal* and *talC3*:

$$f(F6P\_GAP\_TAL) = f(S7P\_E4P\_TAL) \quad (8)$$

And, equation (9) is deduced from metabolite *S7P*:

$$f(S7P\_R5P\_TKT) = f(S7P\_E4P\_TAL) \quad (9)$$

Metabolite *S7P* is only produced by reaction *S7P_R5P_TKT* in the kinetic model, and it is only produced by reaction *TKT1* in the constraint-based model. Thus, in order to further constrain the constraint-based model, the flux of *S7P_R5P_TKT* (kinetic model) can be used as follows to bound the flux *TKT1* (constraint-based model):

$$\begin{aligned} L(TKT1) &= (1-d)\cdot c\cdot f(S7P\_R5P\_TKT) \\ U(TKT1) &= (1+d)\cdot c\cdot f(S7P\_R5P\_TKT) \end{aligned} \quad (10)$$

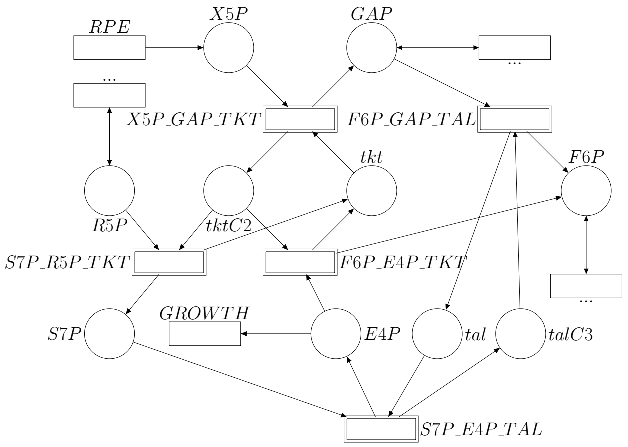

Fig. 5. Petri net representation of the **Subnetwork 3** in the kinetic model. Reactions with a "..." as ID denote multiple reactions. If an arrowhead points to a place, one or multiple reactions produce the metabolite; if an arrowhead points to a transition, the metabolite is consumed by multiple reactions, and if the arrow is bidirectional one or multiple reactions consume or/and produce the metabolite.

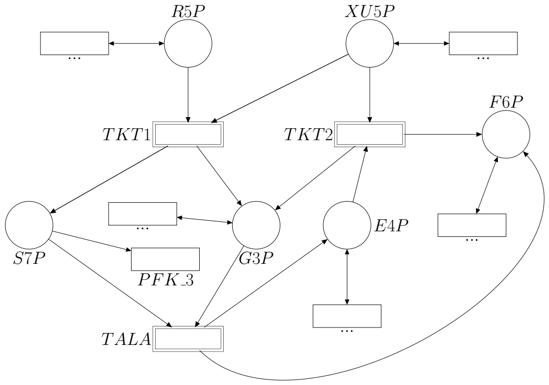

Fig. 6. Petri net representation of the **Subnetwork 3** in the constraint-based model.

On the sequence of kinetic bounds implemented

In the main text, kinetic bounds were implemented according to Table 1. To check the effect of the order in which bounds are implemented, Figure 7 shows the number of dormant reactions and the citramalate production flux under three different orders (the level of uncertainty is 10%). In particular, the implementation order for the results reported in Figure 7 is:

- Figure 7(a): *SUCOAS, PGI, GND, FUM, SUCDi, PDH, G6PDH2r, PYK, TKT1, PFK, PGK, ICDHyr, CS, FBA, PGL, PGM, RPE, AKGDH, RPI, ENO, PPS, ACONTa, PPC, PPCK, FBP, GAPD, ME1, ACONTb, TPI.*
- Figure 7(b): *PPS, SUCDi, FUM, RPI, CS, GAPD, G6PDH2r, ENO, PPC, RPE, PGM, SUCOAS, PGK, TPI, PDH, TKT1, GND, PYK, PPCK, ME1, ACONTb, PGI, PGL, ACONTa, FBA, FBP, ICDHyr, PFK, AKGDH.*
- Figure 7(c): *PPCK, SUCOAS, PGL, GND, TPI, PYK, RPI, ENO, FBA, AKGDH, PGM, GAPD, PGI, FBP,*

ICDHyr, G6PDH2r, ACONTb, PPC, SUCDi, FUM, PPS, PFK, ME1, RPE, PGK, PDH, CS, TKT1, ACONTa.

Note that the effect of permuting the order of implementation of the kinetic bounds in the citramalate-producing iML1515 model neither affected the final citramalate flux nor the number of dormant reactions. However, it did alter the specific point at which a significant activation of previously dormant reactions and a notable reallocation of fluxes occurred.

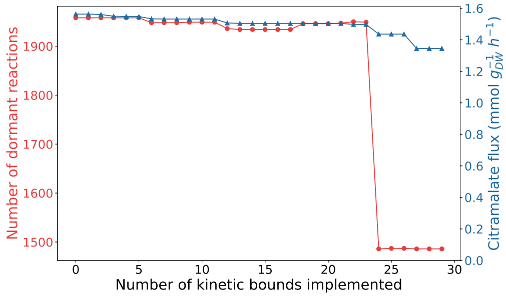

(a)

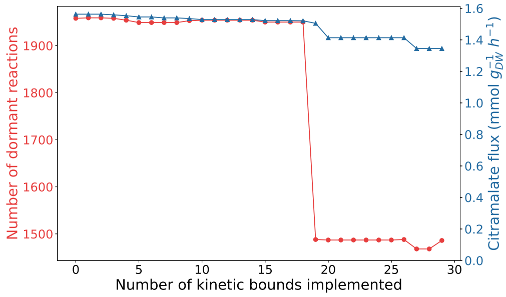

(b)

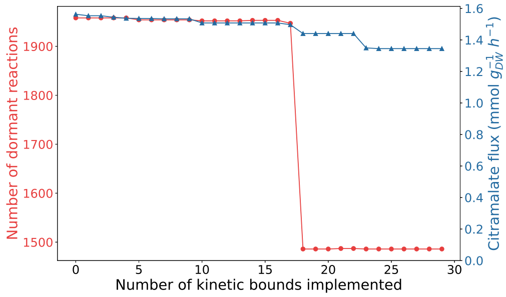

(c)

Fig. 7. Dormant reactions (red line) and citramalate production flux (blue line) under three different randomized orders.

A finer sampling on the uncertainty: Original model

In order to gain insight into the dependence of the number of dormant reactions and the changes in the variability of the reactions with respect to the uncertainty level, we performed a finer analysis on the iML1515 model. The heatmap in Figure 8 represents the number of dormant reactions of the model with respect to the number of kinetic bounds implemented and the uncertainty level, which ranges from 1% to 10%.

This finer analysis showed that when the number of kinetic bounds implemented is high and the uncertainty level is low, the number of dormant reactions in the iML1515 model decreases. Higher uncertainty levels are related to a less constrained model where the addition of kinetic bounds has less effect on the number of dormant reactions.

This is consistent with the intuition that low uncertainty levels constrain the model in such a way that a redistribution of fluxes is necessary. Conversely, higher uncertainty values in the kinetic bounds imply a less restricted model that allows for a higher number of kinetic bounds implemented before a fall in the number of dormant reactions occurs.

Figures 9 and 10 are complementary to the previous analysis. The growth rate (see Figure 9) remains independent of the uncertainty levels when the number of kinetic bounds implemented is less than 13. However, above this value, the growth rate tends to decrease as the uncertainty level diminishes. When 13 kinetic bounds are implemented in the specified order, there is a notable change in the state of the model for lower uncertainty levels. This change in the behavior of the model is also observed at the same point in Figure 8.

Figure 10 shows the same behavior as Figure 8: low levels of uncertainty and high number of kinetic bounds implemented in the iML1515 model limit the model more tightly and impose the reallocation and activation of metabolic routes that originally carried zero flux, which is illustrated by a higher number of reactions increasing their flux variability in relation to the original model.

A finer sampling on the uncertainty: Citramalate-producing model

We reproduced the same analysis with the citramalate-producing model, see Figures 11, 12, and 13, which showed similar trends to the ones illustrated in Figures 8, 9, 10. Again, low uncertainty levels in the kinetic bounds and higher numbers of kinetic bounds implemented imply a lower number of dormant reactions and a higher number of reactions with increased variability. Figure 12 shows how the solution of the problem gets more restricted as the uncertainty level decreases and the number of kinetic bounds implemented increases.

Additional file 1 — Mapping_Kinetic_Stoc.xlsx

This spreadsheet contains the mapping of the reactions between the genome-scale model and the reactions present in the kinetic model ("Mapping" sheet). "flux bounds" sheet contains the upper and lower bounds for each reaction in the genome-scale model that have equivalents in the kinetic model. "kinetic_fluxes_original" reports the fluxes of the reactions in the kinetic model after performing a simulation to reach the steady state. The units of the fluxes are expressed in $mM\ s^{-1}$. Finally, "kinetic_fluxes_citra" reports the kinetic fluxes after a simulation of the citramalate-producing kinetic model until the steady state is reached. The units of the fluxes are expressed in $mM\ s^{-1}$.

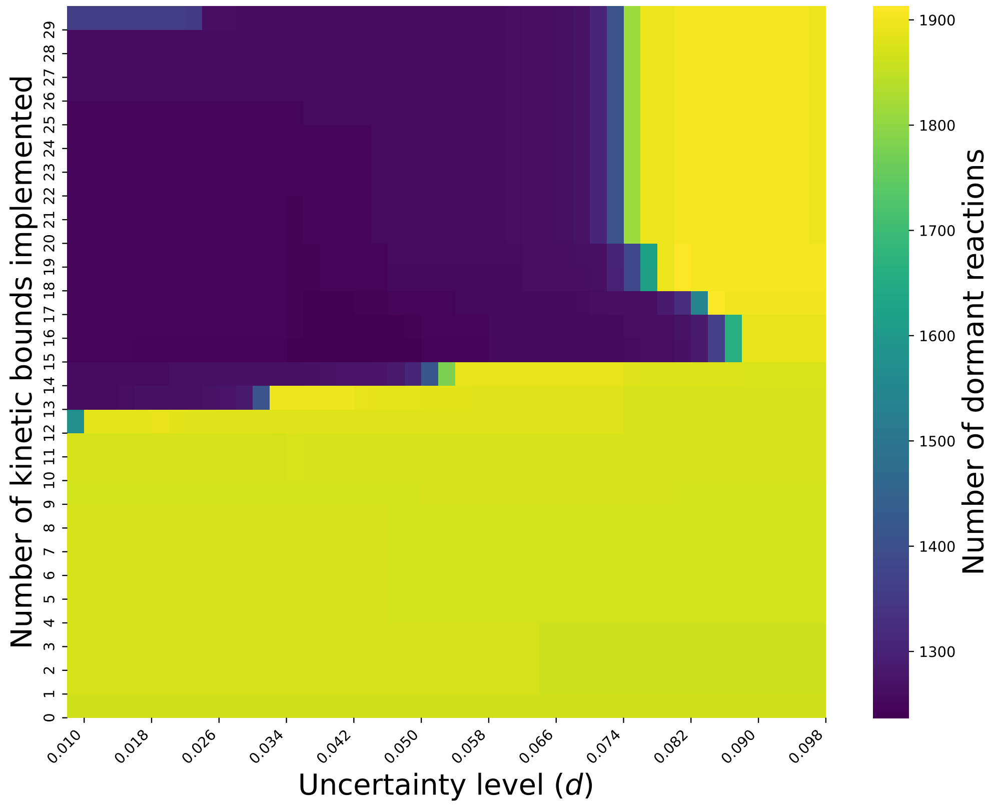

Fig. 8. Number of dormant reactions as a function of the number of kinetic bounds implemented in the iML1515 model and the uncertainty level with which the bounds were integrated (ranging from 1% to 10%).

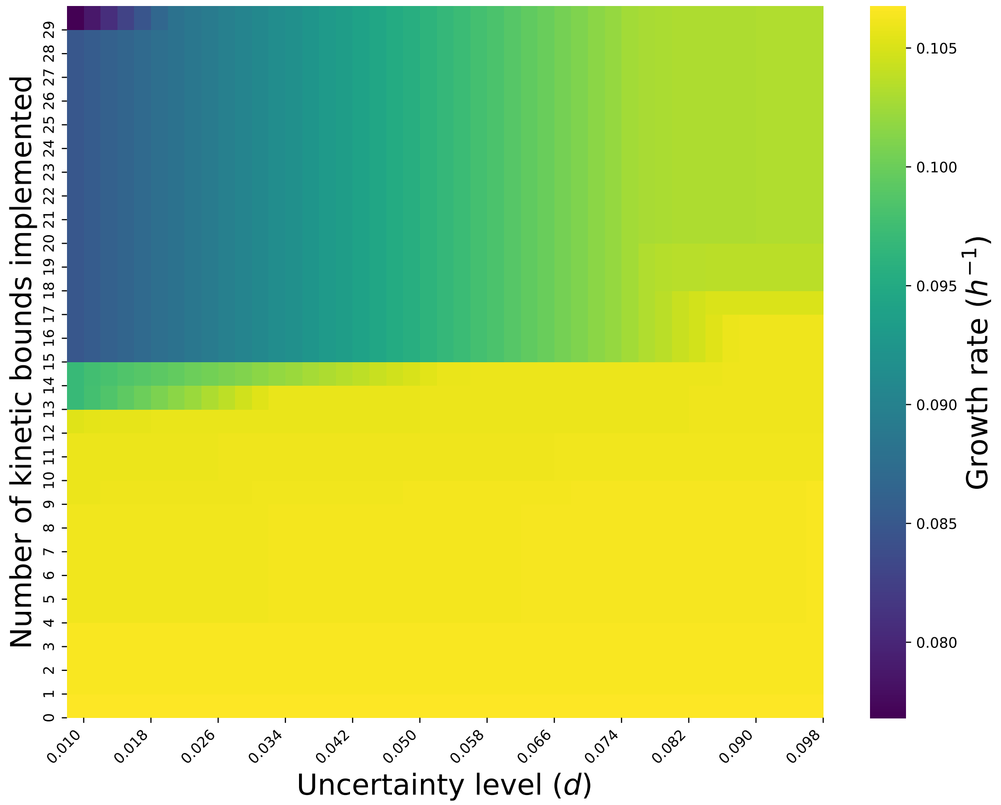

Fig. 9. Growth rate (objective function) as a function of the number of kinetic bounds implemented in the iML1515 model and the uncertainty level with which they were integrated (ranging from 1% to 10%).

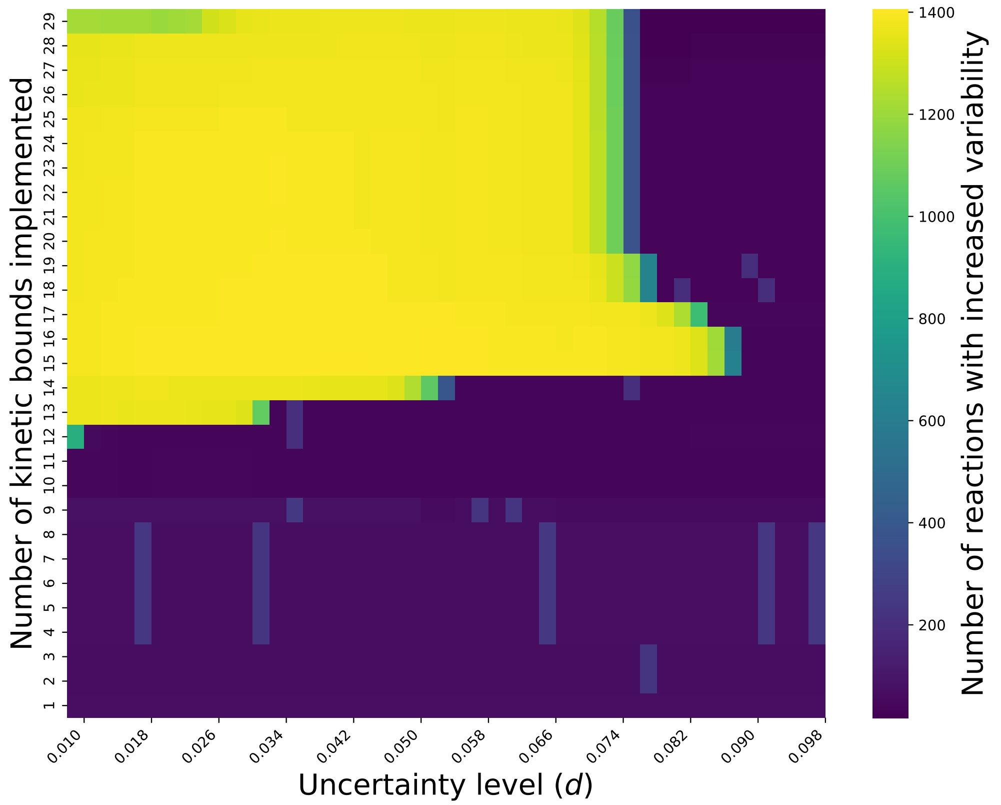

Fig. 10. Number of reactions with increased variability as a function of the number of kinetic bounds implemented in the iML1515 model and the uncertainty level with which the bounds were integrated (ranging from 1% to 10%).

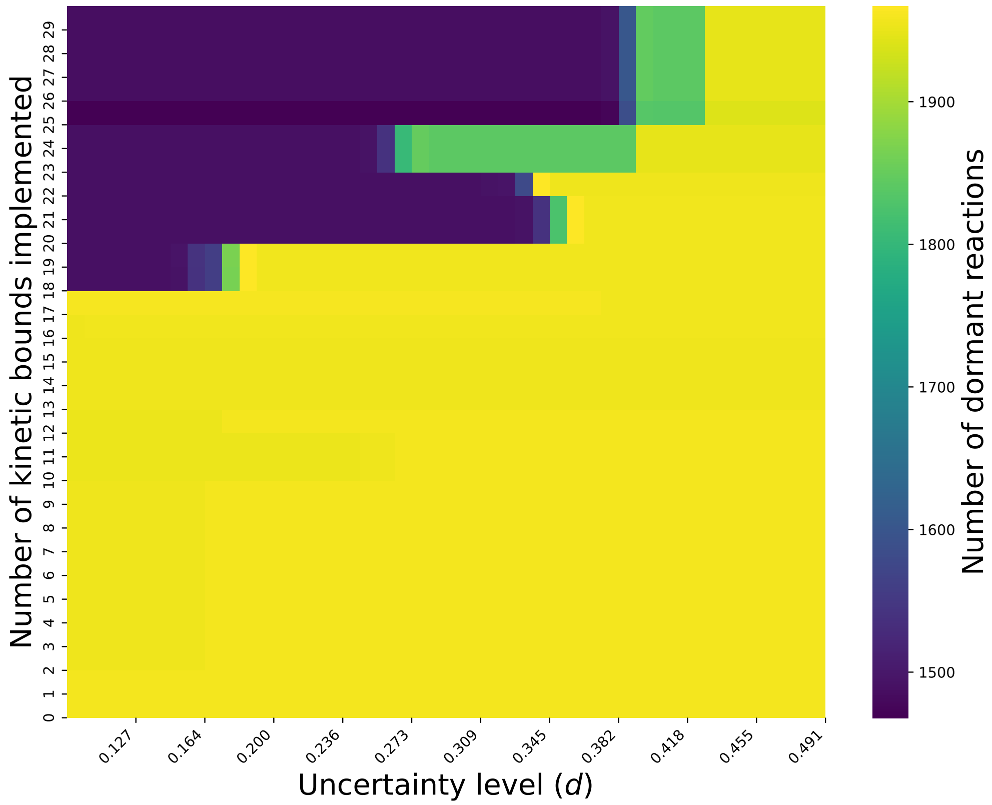

Fig. 11. Number of dormant reactions as a function of the number of kinetic bounds implemented in the citramalate-producing iML1515 model and the uncertainty level with which the bounds were integrated (ranging from 10% to 50%).

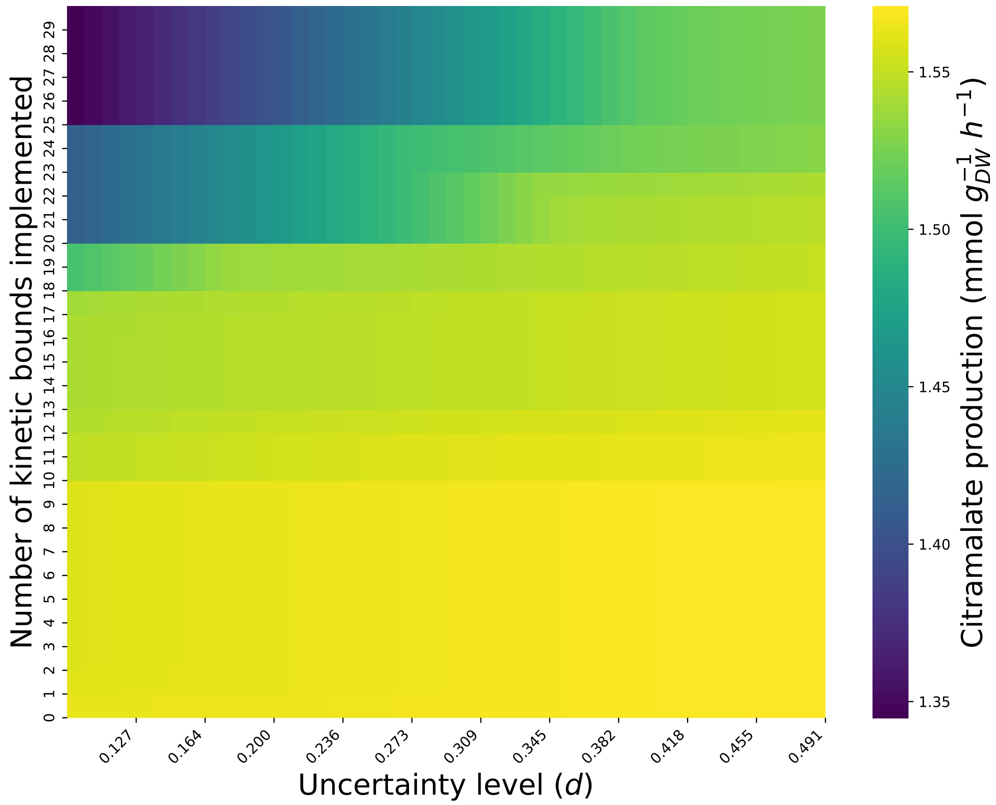

Fig. 12. Growth rate (objective function) as a function of the number of kinetic bounds implemented in the citramalate-producing iML1515 model and the uncertainty level with which they were integrated (ranging from 10% to 50%).

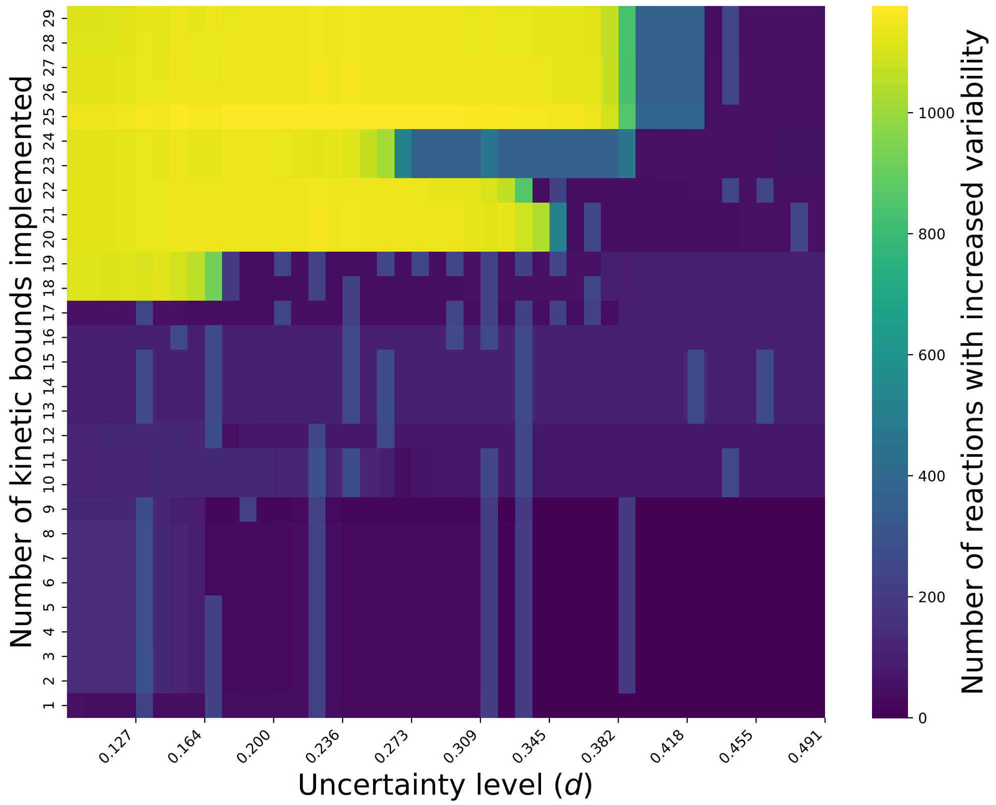

Fig. 13. Number of reactions with increased variability as a function of the number of kinetic bounds implemented in the citramalate-producing iML1515 model and the uncertainty level with which the bounds were integrated (ranging from 10% to 50%).

ID	*name*	*kinetic flux original model (mM s-1)*	*kinetic flux + citramalate (mM s-1)*
1	PGI	0.168	0.170
2	PFK	0.184	0.197
3	FBA	0.184	0.197
4	TPI	0.184	0.197
5	$GAPD$	0.388	0.427
6	PGK	0.388	0.427
7	PGM	0.365	0.426
8	ENO	0.365	0.426
9	PYK	0.158	0.178
10	$G6PDH2r$	0.059	0.059
11	PGL	0.059	0.059
12	GND	0.042	0.040
13	RPE	0.017	0.027
14	RPI	0.025	0.014
15	$TKT1$	0.011	0.013
16	FBP	0.00012	0.00012
17	PPC	0.079	0.062
18	$PPCK$	0.111	0.044
19	PPS	$1.5e-5$	$8.8e-6$
20	$ME1$	0.022	0.032
21	PDH	0.383	0.257
22	CS	0.226	0.039
23	$ACONTa$	0.226	0.039
24	$ACONTb$	0.226	0.039
25	$ICDHyr$	0.128	0.024
26	$AKGDH$	0.111	0.024
27	$SUCOAS$	0.111	0.024
28	$SUCDi$	0.210	0.039
29	FUM	0.210	0.039

Table 1. Reactions whose flux in the constraint-based model was limited to the kinetic bounds computed from the values in the second and third columns depending on the model studied. The kinetic flux values come from the steady state simulation of the kinetic model. Note that the names of the reactions correspond with the ones in the constraint-based model. Some of the names are different in the constraint-based model and the kinetic model, see Additional file 1 for a mapping of reactions between models.
